# Meeting of Physicians of Tyler, and Resolutions on Death of Dr. Starley

**Published:** 1888-01

**Authors:** 


### MEETING OF THE PHYSICIANS OF TYLER, TEXAS.

*Resolutions on the Death of Dr. S. F. Starley.*

A meeting of the regular physicians of Tyler was convened in the office of Dr. A. A. Lyon, December 30, 1887, to take action relative to the death of their late associate, Dr. S. F. Starley.

The meeting, which was largely attended, was organized by calling Dr. E. Jones to the chair and the appointment of Dr. Lyon as secretary. The president briefly stated the object of the meeting to be as above indicated, whereupon, on motion, a committee of three was appointed by the president to prepare a memorial paper and resolutions suited to the occasion. This committee consisted of Drs. A. A. Lyon, A. L. Montgomery and Irvin Pope.

The meeting then took recess, and re-convened later, to hear the report of committee, which was as follows :

Your committee, to whom was referred the duty of preparing a memorial paper, touching the death of our late associate, Dr. S. F. Starley, respectfully beg leave to submit the following :

IN MEMORIAM.

Died, in the city of Tyler, December 19, 1887, Silas F. Starley, M. D. Dr. Starley was born September 5, 1824, in Autauga county, Alabama, and was, therefore, in the 64th year of his age at the time of his death. He removed to Texas at an early age, and settled with his parents at Nacogdoches in 1837. He was educated at Hayneville Academy, and was graduated in medicine from the Medical Department, University of Louisville. His entire professional life was spent in the State of Texas. He had resided and practiced his profession in Cherokee county, Springfield, Fairfield, Corsicana and Tyler.

He was twice married, and had born to him thirteen children, eight of whom survive him. He was a member, first of the Methodist Episcopal, but in 1886 connected himself with the Protestant Episcopal church, in the communion of which he died. In his church relations he was ever faithful and consistent.

As a physician and surgeon Dr. Starley stood in the front rank of the profession in Texas, and has contributed largely to the development of medical science in this State. He was an active and energetic member of the Texas State Medical Association, and was elected its president in 1882, and presided at its session in 1883. He was also an honorary member of the American Medical Association. At different periods in his professional career, he was a conspicuous and valued contributor to various medical journals of this country, including the *North American Medico-Chirurgical Review*, the *New Orleans Medical and Surgical Jonrnal*, the *Southern Journal of Medical Sciences*, *Medical and Surgical Reporter*, *Daniel's Texas Medical Journal*, and others, besides numerous articles read before the State Medical Association.

In his relations, personal and professional, to his medical brethren he was ever affable, generous, courteous and kind, always ready, and with apparent disregard of self, to assist a brother physician in any way that he could ; as a citizen and member of society, he at all times, met in full measure the demands of his station. Therefore be it

*Resolved,* That it is the sense of this meeting that in the death of Dr. Starley the medical profession of Tyler and Texas have lost one of its most valuable and accomplished members, and the community one of its best and most exemplary citizens, the absence of whose benign influence will be sensibly felt wherever he was known, and we, his associates, a brother and friend whose departure we sincerely mourn.

*Resolved,* That we extend to the family of the deceased our heartfelt sympathies in this their sad bereavement ; that a copy of this preamble and resolutions be forwarded to them and also furnished the Waco *Press* and DANIEL'S TEXAS MEDICAL JOURNAL, with a request to publish.

A. A. LYON, M. D., Chairman,
A. L. MONTGOMERY,
IRVIN POPE,
Committee.

The report, as submitted, was unanimously adopted, and the meeting adjourned *sine die.*

A. A. LYON, M. D.,
Secretary.

---

LOCATING THE ASSOCIATION.—As a result of the article on this subject in our last issue, we have received numerous letters. The censensus of opinion, as far as it has been ascertained, is decidedly in favor of a permanent place of abode. *Per contra.* we have had the opinion of some of our ablest and most sagacious members, who oppose it, and submit strong arguments to show that such a step would be fatal to the Association; meantime, our object having been to set the profession to thinking on the subject, so as to be prepared for intelligent action, should it be proposed, we leave the matter *sub judice.*

